# Neurological, Extracardiac, and Cardiac Manifestations of Ebstein’s Anomaly Along With its Genetics, Diagnostic Techniques, Treatment Updates, and the Future Ahead

**DOI:** 10.7759/cureus.35115

**Published:** 2023-02-17

**Authors:** Muhammad Farhan, Priyadarshi Prajjwal, Valleru P Sai, Obed Aubourg, Tappa Ushasree, Herson S Flores Sanga, Arzita Diandra D Fadhilla, Mohammed Dheyaa M Marsool, Nazmun Nahar, Sayantika Ghosh

**Affiliations:** 1 Department of Medicine, College of Medicine, Ajman University, Ajman, ARE; 2 Department of Neurology, Bharati Vidyapeeth University Medical College, Pune, IND; 3 Internal Medicine, Sri Devaraj Urs Medical College, Kolar, IND; 4 Internal Medicine, University of Montreal, Quebec, CAN; 5 Internal Medicine, Ivano-Franskivsk National Medical University, Ivano-Franskivsk, UKR; 6 Cardiology, Hospital Nacional Carlos Alberto Seguin Escobedo, Arequipa, PER; 7 Internal Medicine, Universitas Abdurrab, Pekanbaru, IDN; 8 Internal Medicine, Al-Kindy College of Medicine, University of Baghdad, Baghdad, IRQ; 9 Internal Medicine, Comilla Medical College, Comilla, BGD; 10 Internal Medicine, Georgetown University, Washington DC, USA

**Keywords:** ebstein's extracardiac manifestations, congenital heart disease, ebstein’s anomaly, ebstein’s diagnosis, cardiovascular disease

## Abstract

Ebstein’s anomaly is a congenital heart defect characterized by the displacement of the tricuspid valve, and its leaflets to be malformed. Due to the defect involving the tricuspid valve, there is a reverse flow of blood into the right-sided atrium, which may lead to cardiac hypertrophy and edema of the lower extremities. There is a decreased flow of blood out of the right heart due to reduced right ventricular contractility and tricuspid regurgitation. Children afflicted with this anomaly usually suffer from atrial septal defect and this is usually diagnosed before birth on a routine ultrasound scan. In neonates, cyanosis can be seen due to right-to-left atrial shunting or as a result of severe congestive heart failure. If the infant has pulmonary hypertension, cyanosis is markedly increased as there will be a limitation in pulmonary blood flow. In adults, arrhythmias, cyanosis, and heart failure are seen. The bundle of Kent leads to the formation of an electrical conduction abnormality between the right ventricle and atrium. This leads to a condition commonly known as Wolff- Parkinson-White syndrome in patients. An enlarged spherical heart is usually present on a chest X-ray. ECG changes of Ebstein's anomaly show taller than usual P waves, PR prolongation, and right bundle branch block. There can be certain neurological and extracardiac manifestations too such as hemiplegia, stroke, dysarthria, etc. During fetal life, specifically at 16 and 20 weeks of gestation, the anomaly can be diagnosed via echocardiography. Prostaglandin infusion (PGE1) is given to maintain pulmonary circulation in neonates if cyanosis is seen. In children and adults with congestive cardiac failure due to this anomaly, medical management includes digoxin, beta-blockers, diuretics, and angiotensin converting enzyme (ACE) inhibitors to improve heart failure. Surgical treatment includes valve reconstruction. In this article, we review the pathophysiology, genetics, diagnosis, management, and prognosis of Ebstein’s Anomaly along with a comprehensive discussion on its genetics, neurological manifestations, extracardiac features, and current advancements in treatment.

## Introduction and background

The myogenic muscle organ of the human heart is in charge of preserving the body's blood circulation. Congenital heart abnormalities are caused mainly by an error(s) in the heart's embryological development. With up to one in 200 live births, congenital cardiac abnormalities are a common cause of neonatal deaths [1]. These anomalies have genetic causes, such as mutations, and environmental influences, such as potential teratogen exposure or infections during pregnancy [2].

This anomaly was described in 1866 in a teenage patient of 19 years in Wroclaw, Germany (now Wroclaw, Poland). During that time Wilhelm Ebstein, the person after whom the anomaly is named, was a medical assistant who had just finished his medical career [3]. Some of the symptoms reported by the patient were increased dyspnea and palpitations since infancy and, upon examination, the patient had "marked carotid pulsations synchronous with the heartbeat, percussion over large areas of the chest showed dullness, and systolic and diastolic murmurs". A few days later, the patient died. Some of the findings that Ebstein found at autopsy was the extreme dilation of the right-sided atrium and ventricle along with an abnormal tricuspid valve [4]. The variety of clinical manifestations from infants with severe symptoms to asymptomatic adults makes it the only congenital anomaly with this variety.

Ebstein's anomaly is a congenital heart aberration and is described by the displacement or movement of the apical septal and tricuspid valves. The aforementioned displacement causes the right ventricle to “atrialize”; meaning that the ventricle and the atrium become a single functional chamber, creating an atrial segment. The tricuspid valve also shows a malformation and anterior displacement [5].

## Review

Anatomical correlation

The valves in this abnormality show an unusually deep extension into the right ventricle, and often are larger in size than normal. The valves usually fail, causing the reversal of blood flow, making it flow right back into the right atria again. Such a disturbance, ranging from a mildly to severely turbulent flow, eventually causes the hypertrophic enlargement of the cardiac mass, right heart failure and often results in edema of the liver and lungs [6].

**Figure 1:**
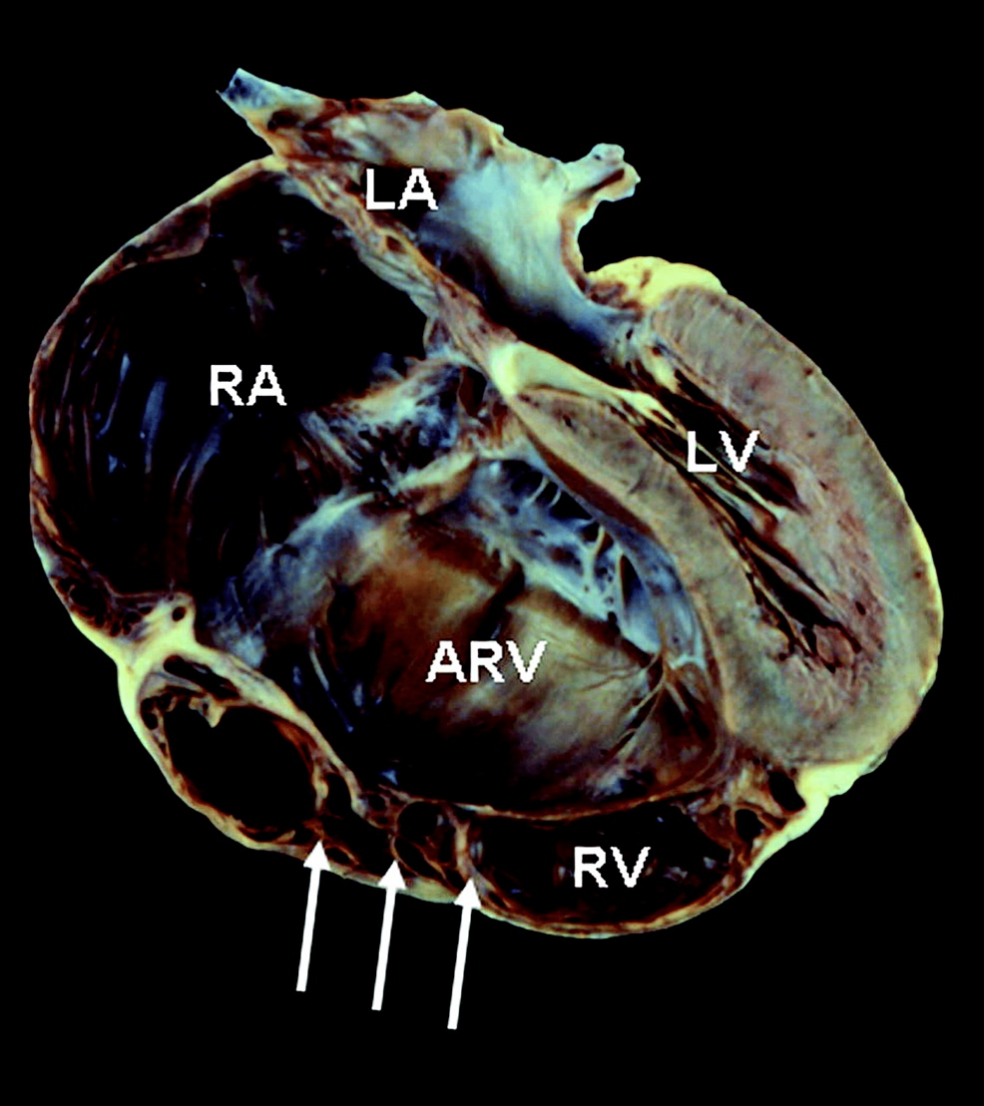
A tricuspid valve with severe Ebstein’s malformation (4-chamber view). The shelf-like posterior leaflet shows a downward displacement, markedly seen with an underlying attachment of free wall by many muscular stumps (indicated with arrows), right ventricle is also markedly dilated, showing “atrilization” (ARV). Right ventricle (RV) is seen as a small portion. And there’s a ventricular septum’s leftward bowing, and dilatation of the right atrium (RA). Image credit: Christine et al. 2007 [9]

The right ventricle in such a condition is usually separated into two parts: the inlet part, which integrates with the atrium of the same side, and the other part which helps in the formation of a functional right ventricle. Also, the true tricuspid valve annulus’ ability to expand, and the existence of a large heart chamber, may be caused by this anomaly. This eventually leads to the detachment of the true tricuspid annulus from the functional right ventricle (Figure 1) [7,8].

Typically, patients may also suffer from an atrial septal defect, which causes deoxygenated blood to flow or be shunted from the right side to the left, past the pulmonary circulation, and then into systemic circulation as blood passes through this defect. Additionally, pulmonary valve stenosis is also possible. The right ventricular cavity itself is typically somewhat tiny in comparison to the normal anatomy, and the right ventricular wall is typically thin [10].

Arrhythmias in this anomaly are not uncommon and may comprise extra electrical pathway abnormalities (such as Wolff-Parkinson-White syndrome) in approximately 15% to 20% of the patients. Atrial fibrillation, which has an increased preponderance with increasing age, can also be seen. The percentage of patients that eventually develop atrial tachyarrhythmias is as high as 30-40% [11-13]. Patients with Ebstein's anomaly also have a heart abnormality called left ventricle non-compaction (LVNC) [14].

Genetics

There’s a lack of comprehensive genetic studies on Ebstein's anomaly; however, some of the newer studies have shown a connection to the *MYH7* gene that is situated on chromosome 14q12. Several studies indicate that this anomaly might be related to sporadic and familial mutations. It has been associated with sarcomeric genes that play a role in myocardial development, including myosin heavy chain 7 (MYH7), transcription factor genes such as *NKX2*, filamin A, *GATA4*, and channel genes such as the V-type voltage-gated sodium channel. The condition is caused by structural protein abnormalities which are important for the contraction of the heart [14].

Approximately 15-29% of patients with this anomaly usually present with findings of left ventricle noncompaction. It is a condition that carries an increased risk of thrombosis and embolism phenomenon, malignant arrhythmias, and left ventricle dysfunction. It has endomyocardial trabeculations that usually rise in number and prominence with time.

In one of the studies determining the link between Ebstein’s anomalies and the *MYH7* gene, 141 patients’ with Ebstien’s anomaly tested for the *MYH7* gene, eight of them had a mutation, while six subjects had LVNC. Therefore, genetic testing and family screening for mutations in *MYH7* are recommended [15].

Another association is with *TPM1*, which encodes α-tropomyosin involved in myocardial contraction and stabilization of actin filaments of the non-muscle cytoskeleton. Finally, a genetic variant named *KLHL26* (p.R237C), which may play an important role in ubiquitin-mediated protein degradation during cardiac development is also related to Ebstein's anomaly.

Pathophysiology

Reduced blood flow out of the right heart is caused by reduced right ventricular contractility and universal tricuspid regurgitation (TR). The valve between the anatomical right atrium (RA) and right ventricle (RV) is absent, which results in inefficient flow (back and forth) between these chambers when they sequentially contract. This is especially true for the RV when this region's myocardium has been extremely weak, frequently leading to inferior dyskinesia. Most frequently, right-to-left shunting occurs along with arterial desaturation, which can be due to the result of this highly inefficient and abnormal flow pattern along with the existence of an atrial septal defect. The RV and the atrium suffer from extreme enlargement as a result of these structural anomalies, which can even occur in young children. This expansion results in increased ventricular mass incompetence and further enlargement of the atrial defect. The degree of cardiac enlargement in some infants and fetuses may even affect how well the lungs develop and work [16,17].

Signs and symptoms

Prenatal

Occasionally, a standard prenatal ultrasound can pick up Ebstein's anomaly as an anomaly in the heart's growth. It is linked to an atrial septal defect, anatomical or functional pulmonary atresia, and in rare cases, an interventricular septum defect. Tachyarrhythmias, hydrops fetalis, pulmonary hypoplasia, heart failure, and cardiomegaly can all result from severe regurgitation of the tricuspid valve and cardiac abnormalities due to Ebstein's abnormality in utero [18,19].

Neonatal

The signs due to Ebstein's anomaly in neonates include 'cyanosis', which is brought on by low levels of oxygen in the blood, 'right congestive cardiac failure', which is brought on by tricuspid valve insufficiency, 'significant cardiomegaly', which is brought on by an enlarging right heart, and 'arrhythmias'. Cyanosis frequently coexists with severe heart failure or with a right-to-left atria shunt. Mild to extremely severe symptoms can be present [5,20].

When a newborn has pulmonary hypertension, the likelihood of cyanosis is noticeably enhanced since the condition further restricts pulmonary blood flow and oxygenation, which in turn causes a higher right ventricular pressure and right-sided failure. Hepatomegaly and a gradual enlargement of the heart may result [21].

Infancy and Adolescence

Typically, the clinical manifestations in infancy and adolescence include 'coughing', 'slow growth', 'exhaustion', 'fast and heavy respiration', 'shortness and difficulty in breathing', and 'rapid heartbeat'. These manifestations may be brought on by the failure of the RV and a reduced ejection fraction of the left ventricle, which raises the heart rate in response to the demands [5].

All of these factors are ultimately impacted by instances of hemodynamic alteration. The clinical profile of these individuals usually depends on the age at which their symptoms appear, the degree of their tricuspid valve displacement, the size and volume of their right ventricle, and the state of the atrial bypass between the right and left sides [22]. Although most affected children with an Ebstein abnormality survive until maturity, some may develop progressive right heart failure [23].

Adulthood

Adult people with minor Ebstein's anomaly may remain asymptomatic or might develop arrhythmias, cyanosis, and worsening right-sided heart failure. These individuals frequently have dilated right atriums and right atrial ventricles, in mild to severe proportions [21]. Most of the individuals having this abnormality usually show findings of abnormal ECGs, regardless of age. Due to the expansion of the right atrium, the ECG may display tall and wide P waves, and low voltage R waves in the leads of V2 and V1 [5].

The Kent bundle, which forms an aberrant electrical connection between the RV and atrium, is also linked to Ebstein's abnormality. The electrical impulse will stray from its normal course as a result of such a beam, increasing the likelihood that the patient would experience Wolff-Parkinson-White syndrome. The supraventricular tachycardia that characterizes this arrhythmia is erratic [5].

Ebstein’s Anomaly in Pregnancy

Most female patients with the abnormality live to adulthood. The pregnancy, maternal problems, and fetal outcomes are all poorly understood. In spite of the presence of cyanosis, one study claimed that when a female with this abnormality reaches her childbearing age, her normal fertility is unaffected [24]. Since it can be challenging to predict all the maternal cardiac risk factors prior to conception, it is advisable to keep track of all the serial modifications in the body and hemodynamic parameters, including the clinical manifestations during pregnancy. There is a need for a proper delivery method to be chosen based on the presentation of the affected females during pregnancy [24].

Diagnosis

The heart's silhouette on the chest radiograph varies from normal to noticeably enlarged or spherical with a tight waist, comparable to that observed in massive pericardial effusions. Vascularization is restricted, and the ascending aorta is frequently nonexistent or very tiny. Infants with symptoms may have "wall-to-wall" and massive cardiomegaly. The vascularization may appear normal in assessing the pulmonary fields or may appear decreased or completely dead due to severe cardiomegaly [11]. An enlarged right atrium is frequently visible. An increased PR interval, right bundle branch block, and taller than normal P waves can all be seen on the ECG. Although a first degree arteriovenous block occurs in nearly half of the individuals, a complete arteriovenous block is uncommon. Older patients have a higher preponderance to experience atrial fibrillation or ventricular fibrillation, while accessory pathway abnormalities account for just 15% of the cases [11,12].

It is recommended to use two-dimensional echocardiography for diagnosis. More recently, 3D echocardiography has been employed to learn more about the subvalvular apparatus and ventricular anatomy. The size and functionality of cavities, septal abnormalities, and other related flaws are also shown by echocardiography. The septal leaflet's apical displacement of 8 mm/m^2^/body surface area is regarded as an important diagnostic sign, as is the absence of delamination at the places where the flaps link to the myocardium inferiorly [24].

Leading-edge details, including muscle or lead connections, or linear connections, are easily determined by 2D and 3D echocardiographic studies. The Great Ormond Street score is often employed to assess newborns [24]. The higher the value of this score, the higher the mortality rate.

In addition to providing more details on the architecture of the heart, MRI is recommended for quantitative measurements of right atrial size, and systolic and diastolic ventricular function [25]. The most accurate way to determine atrial ventricular volume is via axial pictures. The capacity to produce 3D images can also help define the severity of an illness. All patients who would not normally require general anesthesia undergo pre-and postoperative MRIs. MRI is done selectively when general anesthesia is necessary in cases where the patient might be too young or a newborn.

Fetal echocardiography can detect Ebstein's abnormality in the womb between 16 and 20 weeks of pregnancy. It may show cardiac hypertrophy with a right-sided dilated heart accompanied by TR. Since the left ventricular function is often normal during pregnancy, the majority of fetuses handle Ebstein's abnormality successfully. The prognosis is quite poor if there is a progression to hydrops fetalis, which can happen in rare circumstances. Extreme cardiac enlargement can prevent the development of the lungs and results in neonatal respiratory failure [26].

Evaluation of the right-sided ventricular outflow tract is essential because the fetus needs postnatal ductal flow. Supplemental prostaglandin therapy is common to sustain pulmonary circulation if there is an anatomic obstruction or functional pulmonary atresia. Extreme right heart hypertrophy and/or right ventricular outflow obstruction increases the requirement for newborn surgery. Even in the most seasoned hospitals, newborn surgery carries a significant risk, but the prognosis for those who survive is very good. Prenatal counseling should be tailored to each patient and take into account the wide range of severity of Ebstein's defect as well as the possibility that the malformation's severity may worsen during the course of the pregnancy (with progressive right heart enlargement). Depending on the wishes of the parents and the severity of the malformation, selective termination of the pregnancy may be considered [26].

Treatment

Neonates

Because the pulmonary blood flow in the severe neonatal form is majorly dependent on ductus arteriosus, an infusion of prostaglandin is usually given to sustain pulmonary circulation in a symptomatic infant with Ebstein's abnormality. To identify the anomaly, a thorough anatomical evaluation using echocardiography is carried out. Nitric oxide testing can be done to know and assist in the lowering of pulmonary vascular resistance and the prostaglandin withdrawal test is attempted if the RV is free. Surgery is not required if there is no deterioration. But if weaning is unsuccessful, tricuspid valve surgery or a systemic pulmonary artery bypass may be considered [27].

Children and Adults

A cardiologist should routinely check on patients who are asymptomatic or have mild variants of the disease. The individual's heart rate and rhythm should be examined to be aware of any new abnormalities and arrhythmias, and activity tests should be performed to gauge the patient's cardiac function. Standard heart failure therapy is initiated if patients show signs suggestive of right-sided cardiac failure, although there is limited proof that these treatments are effective in people with a history of Ebstein's anomaly [28].

The use of traditional heart failure management therapy, such as beta blockers, diuretics, Angiotensin converting enzyme inhibitors, and digitalis to manage cardiac workload should be properly customized. Recently, nesiritide has also been employed; it enhances hemodynamic activity, boosts sodium excretion, and inhibits the sympathetic overactivity and renin angiotensin aldosterone system. In acyanotic and asymptomatic patients, antibiotics and other drugs are typically not needed. However, if the person has cyanosis or a history of prosthetic valve placement, it is advised to wait before receiving dental treatment [28].

According to guidelines for physical activity, there are no limitations on participating in sports for people with a modest Ebstein abnormality having a heart size that is close to normal with no arrhythmia. Patients are recommended not to exercise in cases of severe, untreated Ebstein's anomalies [29].

Surgical Treatment

On the basis of the individual's age, anatomy, and presenting symptoms, surgical repair is usually implicated. It is advised in cases of severe cyanosis or cardiac failure brought on by malformation of the tricuspid valve. Additionally, if there are symptoms of deterioration, such as growing right ventricular enlargement, cardiomegaly on the chest radiographs, or emergence of premature ventricular beats or ectopics, surgery should be taken into consideration [8].

Patients must each have a unique treatment plan because this condition is difficult to treat. Tricuspid valve reconstruction is the most popular treatment since it is technically feasible but should always be performed by a cardiac surgeon in an equipped hospital [30]. Such a surgery can entail softening the atrial region of the right ventricle or possibly valve repair. The objective of the overall treatment is to create a monocuspid right atrioventricular annulus, therefore tricuspid valve’s anterior flap needs also to be incised, and shifted along the margins of the tricuspid annulus [31].

If the function and size of the RV are sufficient, the biventricular repair is favored as an alternative. The prognosis for symptomatic newborns with this illness is frequently poor due to their adverse pathophysiologic and anatomical circumstances. As a result, successful surgical therapy requires thorough planning and a tailored treatment plan [32].

The majority of newborns with this anomaly do not need surgery. According to the Toronto group, as much as 75% of the neonate population might be cured with conservative management [15]. Those who are relatively stable can be given prostaglandin infusion and supplemental oxygen while being continuously monitored for adequate cardiac output and oxygen saturation. Intubation, heavy sedation with fentanyl (2-4 g/kg/h), paralysis, and the start of prostaglandins are all used to treat unstable individuals. Peripheral vascular resistance (PVR) should be reduced by adjusting ventilation. Inhalation of nitric oxide might be helpful in lowering peripheral vascular resistance. It might be necessary to administer inotrope infusions, such as epinephrine, calcium, and milrinone [15].

After an echocardiography has confirmed the diagnosis, the clinical progression should be cautiously watched. While the prostaglandin infusion is being tapered off, daily echocardiograms should be performed specifically to test for elevated antegrade pulmonary blood. As the PVR falls and the pulmonary blood volume and flow rise over the course of a few days, many infants tend to stabilize and improve [5,15]. Ventilation and prostaglandin infusion gradually decrease as the condition improves [33,34].

Prognosis

With a death rate of up to 85%, this illness is known to have a dismal prognosis in newborns. Up to 48% of pregnancies can pass away suddenly, with congestive heart failure causing hypoxia in 35% of affected live births [35-37]. Given this, numerous cardiac defects, including pulmonary stenosis or atresia, have been linked to the early manifestation of this anomaly. Therefore, the prognosis is typically poorer the earlier the patient gets the condition. The mutation results in larger right atria with a small RV that is hypoplastic, which is caused by the tricuspid valve moving downward [18]. Pulmonary and other valve abnormalities, hepatomegaly, patent ductus arteriosus, and ventricular septum malformations are all risk factors for higher mortality [38,39]. However, people often stay stable and asymptomatic for decades if the anomaly is mild. The majority, however, will experience late arrhythmias at a rate that is comparable to that of patients who have more severe anatomical anomalies [40].

The future ahead

Flap repairs in Ebstein's anomaly are now done much more frequently, which has both decreased early mortality and morbidity of the disease. However, difficulties still persist, particularly for infants with severe myopathy and those who have considerable RV myopathy. Ineffective RV muscle mass plays an important role in restricting exercise tolerance and the emergence of arrhythmias that occur late, even when the heart's function has been surgically restored. Patients with the disease might be able to benefit from the tools in the developing field of "regenerative" cell therapies. By the use of various techniques, such as cell-based technology and organ transplantation from donors, regenerative medicine aims to correct cardiac homeostasis and might result in a better prognosis [41].

The development of de-novo tissue constructions for prognostic and therapeutic purposes for valvular and cardiac structures may also be facilitated by biotechnological methods. The use of cell treatment for a wide range of patients across the world has been pioneered by the field of regenerative heart medicine. Furthermore, compared to the less regenerable cardiac anatomical structures, the science of cardiac regeneration in children has shown a notable rate of cell proliferation in their hearts. For instance, the fields of pediatric and adult regenerative cardiology have made advancements due to the careful selection of the correct patient population. Many cases of structural heart disease can be treated with reconstructive surgery and can be supplemented with a regenerative adjunct at the right time and with suitable cells (sufficient autologous cells to support functional parenchymal tissue). If novel cell platforms show a secure and successful congenital application in children, the reconstructive surgery’s future won't be constrained by an absence of functional tissues or cardiomyocytes [42].

Neurological and extracardiac associations

Uyan et al. reported a case of cerebellar infarction in an Ebstein’s anomaly patient with an atrial septal defect. They suggested that the cerebellar infarct is due to a paradoxical embolism [42]. A more recent case report published by Bowmeester et al. in 2020 also suspected paradoxical embolism as the cause of stroke in a patient with Ebstein’s anomaly. They reported a presentation of sudden onset of dysarthria and left-sided hemiplegia during straining on the toilet [43]. In young patients, ischemic stroke can be caused by a paradoxical embolus [44,45]. Although it is not commonly diagnosed as the cause of arterial ischemia, paradoxical embolus will be more recognizable in suspected embolism patients. The existence of right to left shunting can potentially lead to a paradoxical embolism [46]. Emboli originating from the vein can bypass the normal filtering process in the lungs and enter directly into the systemic circulation. This is possible because of a right to left cardiac shunt, which can result from either a congenital heart defect or higher pressure in the right atrium compared to the left, in which blood flow travels across a patent foramen ovale [44]. Although paradoxical emboli seldom reach the coronary, renal, or splenic arteries, they almost always lodge in the brain and limb circulations [47].

Ebstein’s anomaly is linked to a few extracardiac malformations (ECMs). ECMs are frequently present in fetuses with congenital heart defects [47]. The majority of the problems found are primary malformations, which are typically assumed to develop early in gestation, a remote time period linked to varied maternal recollection. Sieber reported four patients with Ebstein’s anomaly of the tricuspid valve and additional, extracardiac malformations. In the first patient, Ebstein’s anomaly came with hypertelorism, medially rotated hands, a cleft uvula, low-set posteriorly rotated ears, and epicanthal folds. The infant also had cerebral palsy with spastic diplegia, and convulsions, and was deaf and blind. Several external anomalies were noted in the second patient, including a parietal bossing, hypotelorism, beaked snout, thin anterior skull, arachnodactyly, and camptodactyly. The infant died from lung congestion, anasarca, ascites, and bronchopulmonary dysplasia in addition to having chronic renal, hepatic, and cardiac failure. The third patient had articulated eight ribs with the sternum towards the right side, a bilobed uvula, and a short neck. The left kidney and ureter were absent in the fourth patient [48].

## Conclusions

An extremely high prevalence of arrhythmias characterizes Ebstein's anomaly. Due to the possibility of severe tachyarrhythmias, this condition is frequently referred for evaluation, commonly using echocardiography. The underlying embryological abnormalities are the cause of the majority of arrhythmic processes. They can happen in any, from prenatal age as a part of a general or abnormal hemodynamic picture that may be detrimental to the development, to adulthood revealing the diagnosis for the first time. Despite the availability of technology and our understanding of the causes, these patients remain difficult to manage with current electrophysiological and surgical methods. A thorough preoperative electrophysiologic examination and subsequent ablation and correction of the structural abnormality are required to prevent and correct the associated arrhythmias successfully. As with any type of congenital heart disease in adults, there is a need to focus on and address atrial fibrillation management, including its risk stratification to prevent sudden cardiac death among individuals.
